# The Federal and State Narcotic Laws

**Published:** 1915-08

**Authors:** Henry W. Hill

**Affiliations:** Buffalo


### The Federal and State Narcotic Laws.

HENRY W. HILL, LL. D., Buffalo.

Some confusion has arisen as to the provisions of the Federal and State laws relating to the sale of narcotics.

The Act of Congress approved December 17th, 1914, which took effect on March 1st, 1915, known as the Harrison Act, relating to the production, importation, manufacture, compounding, sale, dispensing or giving away of opium or coca leaves, their salts, derivatives or preparations and requiring the registration of all persons dealing therein as well as those giving prescriptions for the use of the same, by its terms relates to the whole of the United States, the District of Columbia, the territory of Alaska, the territory of Hawaii, the insular possessions of the United States to the canal zone. The Act is primarily one of registration and the making and preservation of records of the sale, distribution, dispensation and possession of preparations and remedies, containing more than 2 grains of opium, or more than 1/4 of a grain of morphine, or more than 1/8 of a grain of heroin, or more than 1 grain of codeine or the salts, or derivative of any of them in 1 fluid ounce, or if solid or semi-solid preparation in one avoirdupois ounce, or to liniments, ointments, or other preparations which are prepared for external use only, except liniments, ointments, and other preparations which contain cocaine or any of its salts or alpha or beta eucaine or any of their salts or any synthetic substitute for them; provided, that such remedies and preparations are sold, distributed, given away, dispensed, or possessed as medicines and not for the purpose of evading the intentions and provisions of this Act. The provisions of this Act shall not apply to decocainized coca leaves or preparations made therefrom, or to other preparations of coca leaves which do not contain cocaine.

The Act makes it unlawful for any person not registered under it or who has not paid the special tax to have in his possession or under his control any of the aforesaid drugs and no pharmacist is permitted to sell, dispense or distribute any of the aforesaid drugs to a consumer, except in pursuance of a written prescription, issued by a physician, dentist or veterinary, duly registered under such act, dated on the day signed, which prescription shall be preserved for two years and shall be filed in such way as to be easily accessible to inspection by Federal Officers. A separate file of all such prescriptions must be kept by each druggist and apothecary.

The Commissioner of Internal Revenue with the approval of the Secretary of the Treasury may make all needful rules and regulations for carrying the provisions of the Act into effect.

The Commissioner of Internal Revenue has ruled that "a physician, dentist, or veterinary surgeon can make use of any prescription blank, provided the same is properly dated and signed and has indicated thereon the physician's address, his registry number, and the name and address of the person for whom such prescription is written. The government does not furnish a form upon which prescriptions may be written and the special order form can not be used for this purpose."

He has also ruled that "Only original prescriptions can be filled by druggists and apothecaries and cannot be refilled without violating the law."

The Act expressly provides that it shall not impair, alter, amend, or appeal any of the provisions of the Act of Congress approved June thirtieth, nineteen hundred and six, entitled "An Act for preventing the manufacture, sale, or transportation of adulterated or misbranded, or poisonous, or deleterious foods, drugs, medicines, and liquors, and for regulating traffic therein, and for other purposes," and any amendment thereof, or of the Act approved February ninth, nineteen hundred and nine, entitled "An Act to prohibit the importation and use of opium for other than medicinal purposes," and any amendment thereof.

The constitutionality of the Pure Food and Drugs Act enacted by Congress on June 30th, 1906, has been upheld by the Supreme Court of the United States in various cases.

As already stated the Act of Congress approved December 17, 1914, in relation to the sale and use of narcotics that is opium, coca leaves or the sales of derivatives thereof in express terms applies to the United States and the Collectors of Internal Revenue are charged with its enforcement. Physicians and pharmacists are required to register under it, pay the annual tax imposed, use the order blanks prescribed by the Commissioner of Internal Revenue with the approval of the Secretary of the Treasury, and pharmacists are prohibited from selling, dispensing or distributing any of said drugs to a customer except under a written prescription of a duly registered physician, dentist or veterinary surgeon, and such prescription should be dated on the day when signed by the said physician, dentist or veterinary surgeon and preserved for a period of two years, accessible to inspection by Federal Officers. Other provisions are made in detail as to manufacturers and jobbers selling to dealers that need not be described. Furthermore the Commissioner of Internal Revenue with the approval of the Secretary of the Treasury may make all needed rules and regulations for carrying the provisions of the Act into effect.

The Federal law is a complete code and was intended to provide all the law that is necessary on the subject of narcotics. In fact it is all the law in force in some parts of the

United States. However, it recognizes the fact that there may be state and territorial laws or municipal ordinances regulating the sale, prescribing, dispensing, dealing in or distributing such drugs and such local laws will undoubtedly be held enforceable, where they are not in conflict with the Act of Congress. The states are empowered "to make regulations concerning the same subject matter, reasonable in their terms and not in conflict with the Acts of Congress." That brings us to the consideration of the New York Statutes. Are these in conflict with the Act of Congress on this same subject? Are they reasonable?

Under authority given to the Commissioner of Internal Revenue, he has prescribed rules and regulations in detail, some of which were incorporated by Chapter 327 of the laws of 1915, in the Public Health Law of this State, thus producing a duplication of requirements and reports, wholly unnecessary and intended to harass and annoy pharmacists without serving any useful purpose.

An attempt has been made by amending sections 245, 246, 247, 248, 249, 249-a and 249-d of the New York Public Health Law to harmonize those provisions with the provisions of the Act of Congress hereinbefore referred to and the rules and regulations adopted thereunder so as to avoid conflict.

The introduction of the bill which became chapter 327 of the laws of 1915, is an admission that the two laws were in serious conflict and it is quite possible that they have not yet been fully harmonized.

The Commissioner of Internal Revenue, with consent of the Secretary of the Treasury, has . . . the. . power conferred upon him to . . . make such additional rules and regulations as he may deem necessary to carry into effect the Act of Congress and those rules and regulations, having the authority of law, are likely still to complicate the conditions under which pharmacists now practice their profession.

The conflicts heretofore existing in relation to the forms and requirements of order blanks prescribed by the State and by the Federal Government, which superceded the former and which now have the force of law, is one of the striking evidences of conflict between the two statutes.

Another is the interpretation by the Commissioner of Internal Revenue to the words "dispense," "distribute," or "prescribe," that they are synonymous, which is quite at variance with the popular as well as the technical meaning and use of the word "dispense." Heretofore that has been restricted to the actual delivery of drugs by pharmacists and others to the consumers, but the Commissioner of Internal Revenue enlarges its meaning to include the writing of a prescription by a physician. This cannot fail to produce serious conflict with the wording of the New York statute law, which statute preserved the old well settled etymological distinction in the meaning of the words "dispense," "distribute," and "prescribe." These meanings are well understood and sanctioned by long usage.

This ruling of the Commissioner has the force of law until reversed by the Federal Courts, and supercedes the interpretation heretofore given to the terms of the New York statute. The Federal statute is a complete code in and of itself and assumes the prescription of the physician is a bona fide authorization upon which the pharmacist may rely and act without verifying such prescription as required by chapter 327 of the laws of 1915, amending the foregoing sections of the Public Health Law and by chapter 470 of the laws of 1913, amending the Penal Law in relation to the sale or possession of cocaine or eucaine. This enlarged meaning given to the word "dispense" increases the responsibility of the physician and tends to lessen the responsibility of the pharmacist, as it ought to do. Local authorities have been inclined to hold pharmacists responsible for the acts of physicians who alone come into contact with the patients and are required to make physical examinations before writing prescriptions. Pharmacists have no such information and are not responsible for the diagnosing cases and prescribing remedies. Evidently the framers of the New York law did not anticipate the interpretation that would be given the word "dispensing" by the Commissioner of Internal Revenue and it is doubtful whether the New York Courts would broaden the term to include "prescribing." Therefore wherever it appear there will be a conflict of the two authorities, both asserting jurisdiction over the same matter.

Another variance is that the state laws require before filling the same, verification of the physicians' prescriptions by telephone or otherwise, as to all drugs mentioned the foregoing sections of the Public Health Law and by inquiry as to cocaine and eucaine and their salts, whereas the Federal Act prescribes no such unreasonable requirement but the Collector of Internal Revenue has ruled that "a druggist, when receiving a prescription for any of the drugs coming within the scope of this law, should carefully scrutinize such prescriptions and where he has reason to believe that the same is forged or that the quantity of drug prescribed is unusually large, he should, before filling such prescription, satisfy himself that the same is genuine and properly prepared. Every druggist should know the signature of the reputable, legitimate physicians in his locality, and should he fill a fraudulent prescription he would be liable to prosecution."

The procedure established by this ruling is deemed by the Federal authorities a sufficient protection to the public and might well be adopted by this State and thereby obviate the requirement of verification when it might be impossible to comply with it owing to the fact that the physician cannot be reached, if out on calls or out of town.

The essentials of the prescription prescribed by the Federal Act are accepted and are deemed a sufficient protection to the public and are much more reasonable than is the requirement of the state law, which discredits the validity of the prescription and is a reflection on the medical profession and an unreasonable burden to the profession of pharmacy.

Again the state laws prohibit the refilling of prescriptions (Section 246 of the Public Health Law and Section 1746 of the Penal Law), whereas the Act of Congress contains no prohibition against refilling, although the Commissioner of Internal Revenue has promulgated Art. 12 of his regulations, which does prohibit the refilling of prescriptions and he has also ruled that they may not be refilled. That part of the regulation, prohibiting the refilling of prescriptions, must be observed until reversed or modified by the Federal Courts.

This is a regulation on the part of the Commissioner of Internal Revenue, but it may be within the authority conferred by Sec. 1 of said Act of Congress approved December 17th, 1914.

Furthermore what good reason can there be for an almost complete duplication of requirements to be observed by pharmacists of this state? If the Federal Government is not only to invade but also to usurp the functions of the states as to the public health . . . why burden pharmacists with the attempted observance of two or more separate and more or less conflicting laws on the same subject? These are not likely to be or to remain identical, while the Commissioner of Internal Revenue be given the power to promulgate rules and regulations, which may be modified or changed as often as were the rules, regulations and decisions under the Income Tax Law, so that while attempting to conform to the unsettled policy of the General Government, pharmacists may encounter the over zealous activities of local officers, that recognize no law other than state laws, which are as transitory and as changeable as the capricious activities of certain interests that have been sponsers for the enactment of the several sections of the Public Health Law and their annual modification for half a decade, not wholly for the public welfare.

---

**Solidified Carron Oil.** Pract. Med., March, 1915. Linseed oil eight ounces, white wax two ounces, lime water six ounces. Melt the wax, add the oil, and when mixture is uniform add the limewater, previously warmed. Mix until nearly cold with an egg beater.

